# From abstract to article: Publication outcomes of oral presentations at the ESNR congresses between 2018 and 2022

**DOI:** 10.1007/s00234-026-03970-5

**Published:** 2026-03-13

**Authors:** Ali Murat Koc, Ali Salbas

**Affiliations:** 1 Department of Radiology, Izmir Kâtip Çelebi University, Izmir, Turkey; 2 Department of Radiology, Izmir Atatürk Eğitim ve Araştırma Hastanesi, Izmir, Turkey

**Keywords:** Bibliometric, Citation, Collaboration, Congress abstracts, Neuroradiology, Publication rate

## Abstract

**Purpose:**

This study aimed to evaluate the publication outcomes of oral abstracts presented at the European Society of Neuroradiology (ESNR) congresses between 2018 and 2022 and to identify factors associated with subsequent publication in peer-reviewed journals.

**Methods:**

All oral abstracts presented at ESNR congresses from 2018 to 2022 were identified from the official Books of Abstracts. Publication status was determined through a systematic search of the PubMed/MEDLINE database. For each abstract, subspecialty, study design, and type of institutional collaboration were recorded. Publication rates, time to publication, journal impact factors, and citation metrics were analyzed. Statistical comparisons were performed using chi-square, Kruskal–Wallis, and Mann–Whitney U tests with Bonferroni correction.

**Results:**

Among 241 oral presentations, 114 (47.3%) were subsequently published as full-text articles. The mean time to publication was 16 months, and 90.4% of papers were published within three years after presentation. The most frequent journal of publication was *Neuroradiology*. Publication rates differed significantly by subspecialty, study design, and collaboration type. Studies involving multi-institutional collaboration showed higher publication success, and internationally collaborative studies also received higher citation counts.

**Conclusion:**

Approximately half of the oral presentations at ESNR congresses were published in peer-reviewed journals. Multi-institutional collaboration substantially enhances publication success and academic impact. Encouraging collaboration and supporting research development within the ESNR framework may further improve the visibility and strengthen the academic influence of future studies.

## Introduction

 Scientific congresses serve as key platforms for the dissemination of novel research findings, professional networking, and the establishment of academic collaborations [1]. Beyond their social and educational value, congresses provide researchers with the opportunity to present preliminary results, receive critical feedback from peers, and refine their studies prior to journal submission. Abstracts presented at scientific meetings are therefore often regarded as the preliminary stage of research that may subsequently evolve into full-text publications. The extent to which presented abstracts are later published in peer-reviewed journals is considered an important indicator of the scientific impact and academic productivity associated with a congress [1, 2].

Previous studies have investigated the publication rates of abstracts in several medical specialties [3]. In radiology, the literature has largely focused on the European Congress of Radiology (ECR), interventional radiology meetings such as the Cardiovascular and Interventional Radiology Society of Europe (CIRSE) and the Society of Interventional Radiology (SIR), the European Society of Gastrointestinal and Abdominal Radiology (ESGAR), as well as several national congresses [1, 2, 4–7]. However, to date, no study has specifically examined the publication outcomes of abstracts presented at the European Society of Neuroradiology (ESNR) congress, which represents one of the leading international meetings in the field of neuroradiology.

The aim of the present study was therefore to evaluate the publication outcomes of oral abstracts presented at the ESNR congresses between 2018 and 2022. By analyzing factors such as subspecialty distribution, study design, and patterns of institutional collaboration, this study seeks to provide insights into the scientific dissemination and academic contribution of research presented at ESNR.

## Methods

This study evaluated the publication outcomes of oral abstracts presented at the annual meetings of the ESNR between 2018 and 2022. Abstracts were obtained from the official Books of Abstracts, which are published annually as supplements in the journal Neuroradiology [8–12]. Previous studies have reported that the mean time from congress presentation to full-text publication is approximately 15–18 months, with about 80% of publications appearing within the first three years [5–7, 13]. Accordingly, to ensure an adequate follow-up period, only abstracts from congresses held up to 2022 were included, as data collection was conducted in 2025. Abstracts presented at the 2023 and 2024 congresses were excluded due to insufficient follow-up time for potential publication.

Only oral presentations were included to ensure comparability with previous investigations that primarily analyzed such presentations [1, 6, 13, 14]. A total of 889 abstracts were identified in the ESNR Books of Abstracts between 2018 and 2022. Of these, poster presentations (*n* = 628) were excluded. In addition, abstracts that had been published before the corresponding congress date (*n* = 20) were excluded. Consequently, 241 oral presentations were included in the final analysis.

Two independent radiologists reviewed all oral abstracts and extracted the following data: subspecialty, study design, and level of institutional collaboration. The subspecialties were recorded exactly as listed in the official abstract booklets, namely Brain, Interventional Radiology, Pediatrics, Head and Neck, and Spine. The country of the first author was recorded as provided in the abstract booklet. In addition, the corresponding continent of each country was recorded. For the purposes of geographical classification, Europe was defined to include the 28 member states of the European Union as of 2018, as well as Bosnia and Herzegovina, Belarus, North Macedonia, Moldova, Norway, Russia, Serbia, Switzerland, Turkey, and Ukraine [15]. Study design was classified as retrospective, prospective, or other (e.g., experimental studies, surveys) [6].

Collaboration among co-authors was categorized into three groups based on institutional affiliation: local collaboration (all authors from the same institution), national collaboration (authors from different institutions within the same country), and international collaboration (authors from institutions located in two or more different countries). This classification was adapted from the methodology of previous studies [1].

Only full-text original research articles were included in the analysis; reviews, editorials, case reports, pictorial essays, and letters were excluded. To determine whether the abstracts had been subsequently published, a comprehensive search of the MEDLINE database via the PubMed platform was conducted between September 15 and October 15, 2025 [16]. The search strategy was designed in accordance with previous studies [6, 13, 14]. Initially, the full surname and initials of the first author were searched. If no corresponding publication was identified, a separate search was conducted using the second author’s name. If still no match was found, additional searches were performed using the names of other co-authors listed in the abstract. When more than 20 potential matches were retrieved, key terms derived from the abstract titles were added to refine the search results. Each potential match was cross-checked by both reviewers, and only those articles that were confirmed by consensus to correspond to the original abstract (based on author names, study design, and subject matter) were accepted. Any discrepancies between the two reviewers were resolved by consultation with a third senior radiologist.

For each published article, the following data were collected: title of the publication, name of the journal, date of publication, the journal’s impact factor for that year, and the total number of citations as of October 15, 2025 according to Google Scholar [17, 18]. In addition to total citation counts, annualized citation rates were calculated by dividing the total number of citations by the time elapsed since publication, expressed in years (number of days between the publication date and October 15, 2025, divided by 365.25). The time to publication was calculated as the number of days between the start date of the corresponding congress and the publication date. For studies published online ahead of print, the early online publication date was used for this calculation.

## Statistical Analysis

All statistical analyses were performed using IBM SPSS Statistics (version 26, IBM Corp., Armonk, NY, USA). A p-value < 0.05 was considered statistically significant in all analyses.

Descriptive data were expressed as counts and percentages for categorical variables, and as mean ± standard deviation (SD) or median with interquartile range (IQR) for continuous variables.

Countries with ≥ 10 oral presentations were analyzed separately, and those with fewer were grouped as “Other.” Publication outcomes were compared across countries using chi-square tests and univariable logistic regression analyses, with Italy serving as the reference category because it had the highest number of presentations, provided a stable sample size for comparison, and showed a midrange publication rate, avoiding the selection of a category with outlying values as the reference.

Associations between subspecialty, study design, and collaboration type with subsequent publication were examined using chi-square tests. Pairwise comparisons were additionally performed using chi-square tests with Bonferroni correction to identify differences between specific groups.

The distribution of continuous variables was evaluated using the Shapiro–Wilk test for normality. Because both annual citation and JIF values were non-normally distributed, group comparisons were performed using the Kruskal–Wallis test. Pairwise Mann–Whitney U tests with Bonferroni correction were conducted for post-hoc comparisons where appropriate.

## Results

**Fig. 1:**
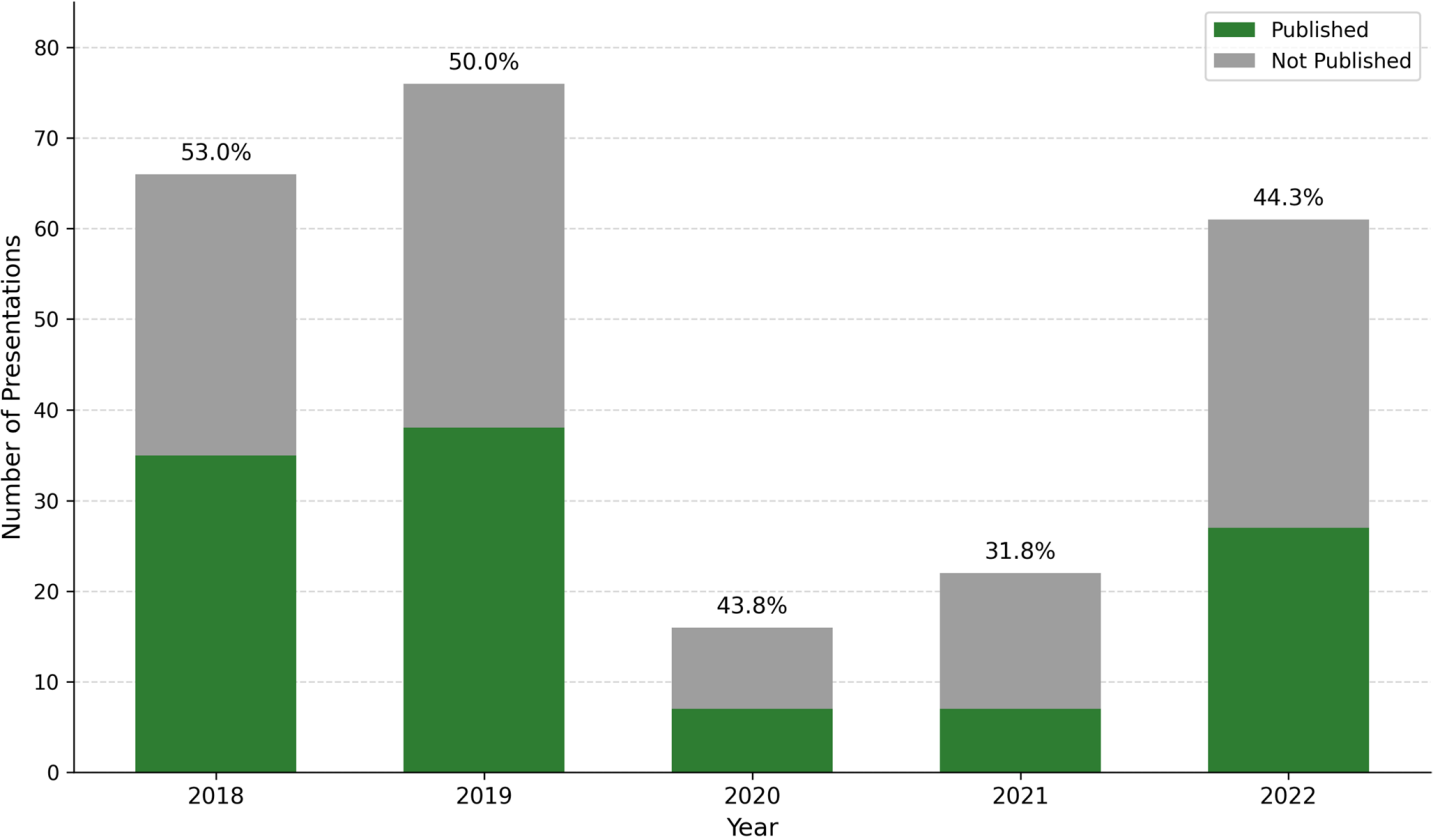
Distribution of oral presentations and publication rates by congress year. Each bar represents the total number of presentations in that year; green segments indicate published abstracts and gray segments indicate unpublished one

A total of 241 oral abstracts presented at the ESNR congresses between 2018 and 2022 were included in the analysis. Among these, 114 (47.3%) were subsequently published as original research articles in journals indexed in PubMed/MEDLINE. Publications received a median of 12.0 citations (IQR, 7.0–26.8; range, 0–231). The mean journal impact factor (JIF) was 4.22 (SD = 2.44; range, 1.2–15.2). The median time to publication was 13.0 months (IQR, 7.1–21.0), with a mean of 16.1 months (SD, 13.8; range, 0.4–58.3 months). Of the 114 published studies, 103 (90.4%) were published within the first three years after presentation (Table 1). The highest number of oral presentations was delivered in 2019 (*n* = 76), followed by 2018 (*n* = 66). Abstracts presented in 2018 and 2019 had the highest subsequent publication rates (53.0% and 50.0%, respectively) (Fig. 1).

**Table 1:** Distribution of time from congress presentation to publication

Publication time	*n*	%	Cumulative %
0–12 months	31	27.2	27.2
13–24 months	39	34.2	61.4
25–36 months	33	28.9	90.4
37–48 months	8	7.0	97.4
49–60 months	3	2.6	100.0
> 60 months	0	0	100.0
Total	**114**	**100.0**	**-**

Time to publication was calculated as the interval between the start date of the corresponding ESNR congress and the publication date. For studies published online ahead of print, the online publication date was used. Percentages were calculated based on 114 published abstracts

The 114 published articles were distributed across 53 different journals. *Neuroradiology* was the most frequent publication venue, accounting for 16.7% (19/114) of all articles, followed by *European Radiology* (7.9%, 9/114) and the *American Journal of Neuroradiology (AJNR)* (6.1%, 7/114). Together, these three journals published 30.7% (35/114) of all articles (Table 2).

**Table 2:** Distribution of published articles according to journals

Journal	*n*	**%**
*Neuroradiology*	19	16.7
*European Radiology*	9	7.9
*Stroke*	7	6.1
*American Journal of Neuroradiology (AJNR)*	7	6.1
*Frontiers in Neurology*	4	3.5
*Journal of Neuroradiology*	3	2.6
*Interventional Neuroradiology*	3	2.6
*PLoS One*	3	2.6
*Other journals (<3 publications)*	59	51.8
**Total**	114	100

Only journals with ≥3 publications are listed individually; all others were grouped as “Others”. The “Others” category consisted of 59 publications distributed across 45 different journals

A total of 30 countries contributed oral presentations to the ESNR congresses between 2018 and 2022. Most presentations originated from Europe (*n* = 201, 83.4%), followed by Asia (*n* = 27, 11.2%). The countries with the highest number of oral presentations were Italy (*n* = 39, 16.2%), Portugal (*n* = 26, 10.8%), and both Germany and the United Kingdom (each *n* = 19, 7.9%).

Country of origin was statistically related to subsequent publication of the abstract (χ²=32.40, *p* < 0.001). Among countries with ten or more presentations, the highest publication rates were observed in the Netherlands (75.0%, 12/16), South Korea (72.7%, 8/11), and Germany (63.2%, 12/19). Publication rates by country are summarized in Table 3.

**Table 3:** Publication rates according to country of origin

Country	Presentations (*n*)	Published *n* (%)	Odds ratio (95% CI)^a^	χ²	*p*
Italy	39	24 (61.5%)	1.00	**-**	**-**
Netherlands	16	12 (75.0%)	1.88 (0.51–6.90.51.90)	0.89	0.344
South Korea	11	8 (72.7%)	1.67 (0.38–7.29.38.29)	0.46	0.497
Germany	19	12 (63.2%)	1.07 (0.34–3.33.34.33)	0.01	0.905
United Kingdom	19	10 (52.6%)	0.69 (0.23–2.10.23.10)	0.42	0.519
Switzerland	10	5 (50.0%)	0.63 (0.15–2.53.15.53)	0.43	0.510
United States	12	5 (41.7%)	0.45 (0.12–1.67.12.67)	1.44	0.230
Spain	14	4 (28.6%)	0.25 (0.07–0.94.07.94)	4.19	0.041^b^
Portugal	26	4 (15.4%)	0.11 (0.03–0.39.03.39)	11.71	<0.001^b^
Russia	11	1 (9.1%)	0.06 (0.01–0.54.01.54)	6.36	0.012^b^
Other	64	29 (45.3%)	0.52 (0.23–1.17.23.17)	2.53	0.112

Countries with ≥10 oral presentations were analyzed separately; those with fewer were grouped as “Other.” *CI* Confidence interval

^a^ Values in parentheses are 95% confidence intervals

^b^ Statistically significant difference compared with Italy (reference) (p<0.05)

The most frequently presented subspecialty was Brain (*n* = 128, 53.1%), followed by Interventional Radiology (*n* = 34, 14.1%) and Pediatrics (*n* = 34, 14.1%). Subspecialty category was significantly associated with subsequent publication outcome (χ²=10.10, *p* = 0.039). The highest publication rate was observed in the Brain category (55.5%). Publication rates by subspecialty are summarized in Table 4. No significant difference was observed in annual citations among subspecialties (*p* = 0.108). Similarly, there was no significant variation in the journal impact factor across subspecialties (*p* = 0.404).

**Table 4:** Publication outcomes, annual citations, and journal impact factors according to subspecialty, study design, and collaboration type

	**Presentations (n)**	**Published n (%)**	**Annual citations**	**JIF**
**Subspecialty**				
Brain	128	71 (55.5%)	3.5 (1.9–6.6)	3.6 (2.8–4.8)
Head & Neck	16	8 (50.0%)	2.5 (1.7–3.4)	3.5 (2.8–3.7)
Interventional	34	15 (44.1%)	5.0 (3.7–9.1)	4.6 (2.8–7.2)
Pediatric	34	12 (35.3%)	2.2 (1.3–3.5)	3.4 (2.7–4.7)
Spine	29	8 (27.6%)	3.0 (0.8–6.2)	2.9 (2.3–3.4)
*p*	-	**0.039**	0.108	0.404
**Study design**				
Retrospective	113	56 (49.6%)	3.4 (2.0–6.3)	3.2 (2.4–5.0)
Prospective	101	53 (52.5%)	3.4 (1.6–5.7)	3.6 (2.8–4.8)
Other	27	5 (18.5%)	4.7 (3.1–5.2)	4.0 (3.3–5.4)
*p*	-	**0.006**	0.491	0.462
**Collaboration Type**				
Local	134	48 (35.8%)	3.1 (1.8–4.8)	3.6 (2.8–4.8)
National	54	34 (63.0%)	3.2 (1.7–7.1)	3.1 (2.7–3.8)
International	53	32 (60.4%)	4.9 (3.0–9.2)	3.8 (2.9–7.0)
*p*	-	**<0.001**	**0.042**	0.145
**Total**	241	114 (47.3%)	3.4 (1.8–5.9.8.9)	3.6 (2.8–4.8.8.8)

Data are presented as number (percentage) or median (interquartile range). Annual citations were calculated as the total number of citations divided by years since publication. JIF, journal impact factor. Citation and JIF data include only the 114 abstracts that were subsequently published as full-text articles. JIF values correspond to the impact factor of the journal in the year the article was published. JIF data were available for 112 publications; JIF values were not available for 2 articles. Significant p-values are shown in bold. The “Other” category in study design includes experimental, computer-based, or methodological studies, as well as abstracts without a clearly defined study design that did not fit into retrospective or prospective classifications

Study design was significantly associated with subsequent publication outcome (*p* = 0.006). The highest publication rate was observed in prospective studies (52.5%) (Table 4). Studies categorized as “Other” had significantly lower publication rates than both retrospective and prospective designs (*p* = 0.007 and *p* = 0.003, respectively), whereas no significant difference was found between retrospective and prospective studies (*p* = 0.772). There was no significant difference in annual citations (*p* = 0.491) or journal impact factor (*p* = 0.462) among study design categories.

Collaboration type was significantly associated with subsequent publication outcome (*p* < 0.001). The highest publication rate was observed in nationally collaborative studies (63.0%), followed by international (60.4%) and local collaborations (35.8%) (Table 4). National and international collaborations showed significantly higher publication rates compared with locally conducted studies (*p* = 0.001 and *p* = 0.004, respectively), whereas no significant difference was observed between national and international collaborations (*p* = 0.939).

There was no significant difference in journal impact factor among collaboration categories (*p* = 0.145). Annual citations differed significantly by collaboration type (*p* = 0.042). Internationally collaborative studies received significantly higher annual citations than locally conducted studies (*p* = 0.026), while no significant differences were found between national–local (*p* = 1.000) or national–international (*p* = 0.480) comparisons.

## Discussion

This study demonstrated that approximately half (47.3%) of the oral presentations delivered at the ESNR congresses between 2018 and 2022 were subsequently published as articles indexed in PubMed/MEDLINE. Moreover, publication outcomes showed notable variation depending on subspecialty, study design, and institutional collaboration, suggesting that multiple factors influence research dissemination within ESNR.

The rate at which congress presentations are subsequently published varies considerably across medical specialties and the nature of the meetings. A comprehensive meta-analysis including more than 300,000 abstracts reported an overall publication rate of 37.3% across all medical disciplines [3]. In radiology-focused research, publication rates reported for international meetings generally range between 33% and 51.8% [6, 19, 20]. For the European Congress of Radiology (ECR), two separate studies analyzing the 2000 and 2001 meetings reported publication rates of 47% and 45%, respectively [13, 14]. Loughborough et al. reported a 43% publication rate for ECR 2010, whereas another investigation of the same meeting found a rate of 51.8% [1, 20]. Such variability, even between studies evaluating the same meeting, likely reflects methodological differences in follow-up and search strategies.

In another study, the Skeletal Society of Radiology (SSR) meeting was reported to have a publication rate of 50.6%, which is relatively high compared with other congresses [21]. This may be partly explained by the inclusion of databases beyond PubMed in that analysis. Evidence from neuroradiology-focused congresses is scarce. To date, only one study has evaluated publication outcomes for the American Society of Neuroradiology (ASNR) 1993 meeting, reporting a 37% publication rate [22]. Given the limited availability of more recent neuroradiology-specific data, findings from other subspecialty meetings may provide additional context for comparison. For example, a study evaluating the ESGAR meetings between 2019 and 2022 found a conversion rate of 52.8%, which is higher than that reported in earlier meetings of the same society [23]. The publication rates reported for other radiology and medical congresses are summarized in Table 5 [1, 5, 6, 14, 19–31]. However, these comparisons should be interpreted with caution, as the heterogeneity in follow-up durations and study periods across the literature may limit direct comparability between different meetings.

**Table 5:** Comparison of publication rates across international radiology and other medical meetings

Congress	Specialty	Publication rate (%)	Database(s) searched	Follow-up period
ASNR (1993)	Neuroradiology	37.0	PubMed	4 yrs
RSNA (1995)	Radiology	33.0	PubMed	5 yrs
ECR (2000)	Radiology	47.0	PubMed	4 yrs 9 months
ECR (2001)	Radiology	45.0	PubMed	4 yrs 9 months
ESGAR (2000-2001)	GI Radiology	39.5	PubMed	3–4 yrs
ESGAR (2019-2022)	GI Radiology	52.8	PubMed	3–6 yrs
ECR (2010)	Radiology	43.0	PubMed	4 yrs 9 months
ECR (2010)	Radiology	51.8	PubMed	5 yrs
ESPR (2013-2015), SPR (2013-2015), IPR (2016)	Paediatric Radiology	46.0	PubMed	5–7 yrs
CIRSE (2012)	Interventional Radiology	46.8	PubMed + Google Scholar	3 yrs
SIR (2012)	Interventional Radiology	44.1	PubMed + Google Scholar	3 yrs
SSR (2010-2015)	MSK Radiology	50.6	PubMed + Google Scholar + Google	2–7 yrs
**ESNR (2018-2022)** **(current study)**	Neuroradiology	47.3	PubMed	3–7 yrs
AAO (2008)	Ophthalmology	57.8	PubMed	6 yrs
ASA (2009)	Anesthesiology	22.0	PubMed + Google Scholar	~3 yrs
AHA (2006-2008) ACC (2006-2008) ESC (2006-2008)	Cardiology	49.7 42.6 37.6	Web of Science	5 yrs
NASS (2009-2011)	Spine Surgery	51.0	PubMed	3–5 yrs
EPOS (2006-2008)	Paediatric Orthopaedic	36.7	PubMed	5 yrs
AAOS (2016-2017)	Orthopedics	71.6	PubMed + Google Scholar	4–5 yrs
AAOS (2022-2023)	Orthopedics	44.0	PubMed + Google Scholar	1 yrs

*AAO*, American Academy of Ophthalmology, *AAOS* American Academy of Orthopaedic Surgeons, *ACC* American College of Cardiology, *AHA* American Heart Association, *ASA* American Society of Anesthesiologists, *ASNR,* American Society of Neuroradiology, *CIRSE* Cardiovascular and Interventional Radiological Society of Europe, *ECR* European Congress of Radiology, *ESC* European Society of Cardiology, *ESGAR *European Society of Gastrointestinal and Abdominal Radiology, *ESNR* European Society of Neuroradiology, *ESPR* European Society of Paediatric Radiology, *EPOS* European Paediatric Orthopaedic Society, *IPR* International Pediatric Radiology, *NASS* North American Spine Society, *RSNA* Radiological Society of North America, *SIR* Society of Interventional Radiology, *SPR* Society for Pediatric Radiology, *SSR* Skeletal Society of Radiology. Sources: Data compiled from the cited references [1, 5, 6, 14, 19–31]

In the present study, the publication rate for oral presentations delivered at the ESNR congresses between 2018 and 2022 was 47.3%. This proportion places ESNR in the upper range of publication outcomes reported for radiology-related international meetings. Differences in reported publication rates across studies may reflect variations in congress scope, methodological design, and data collection period. Unlike larger general radiology meetings such as ECR or RSNA (Radiological Society of North America), the ESNR congress focuses on a narrower subspecialty field and accepts a smaller number of oral presentations each year. For instance, while ECR 2019 hosted approximately 1,900 oral presentations, ESNR 2019 included only 76 [32]. This smaller scale likely allows for a more selective review process, resulting in higher-quality submissions and, consequently, a relatively higher publication rate.

In the present study, *Neuroradiology* was the most frequent journal for ESNR presentations, which is expected since it serves as the official journal of the society. This pattern is consistent with findings from other major radiology meetings, where subsequent publications often appear in the official journals of the respective societies. Previous analyses of ECR abstracts have shown that most subsequent publications appeared in *European Radiology*, whereas approximately one-third of RSNA presentations were published in *Radiology* [13, 20]. Similarly, analyses of ESGAR presentations have shown that most subsequent publications appeared in *European Radiology* [6]. In addition, presentations from the Skeletal Society of Radiology (SSR) meetings were most frequently published in *Skeletal Radiology*, which closely aligns with the congress’s subspecialty focus [21]. The predominance of the journal *Neuroradiology* among ESNR-related publications is an expected finding that may be related both to the journal’s association with the society and to the close overlap between the congress’s scientific scope and the journal’s thematic focus. While such institutional alignment may provide a structural publication advantage and potentially influence conversion rates, it also ensures that specialized research reaches its most relevant audience through established academic channels.

When analyzed by year, the lowest number of presentations and the lowest publication rates were observed in 2020 and 2021, which should be interpreted with caution and may be partly related to the effects of the COVID-19 pandemic as well as to the shorter follow-up time for these more recent congresses. Literature reports that, during the pandemic period, manuscript submissions to journals increased, whereas the overall number of published articles did not show a comparable rise. In addition, the number of studies submitted to conferences markedly declined in 2020–2021 [33]. However, another study demonstrated that individual publication productivity among radiologists increased during the same period, likely due to reduced clinical workload and increased time for academic activities [34]. Our findings may be explained by several factors. The shift to fully virtual or hybrid congress formats may have led organizers to limit the number of sessions, resulting in fewer presentations. In addition, radiology departments experienced major workflow disruptions during the pandemic, with elective studies postponed and research priorities redirected toward COVID-related topics [35]. Moreover, many abstracts presented in 2020 and 2021 may have represented projects that had not yet reached full maturity due to pandemic-related interruptions, and their subsequent publication may have been underestimated because of the limited follow-up duration.

Previous studies have reported mean times to publication of approximately 15.0 months for CIRSE and 16.3 months for SIR meetings, and 18 months for ESGAR congresses [5, 6]. An analysis of a national radiology meeting also reported a mean interval of 15 months [7]. In our study, the mean time to publication was 16.1 months, which is broadly consistent with these findings. However, these earlier investigations focused on congresses held more than a decade ago. With the increasing use of online submission systems and early online publication, a shorter publication interval might have been expected. The comparable time observed in our study may reflect residual effects of the COVID-19 pandemic, which temporarily disrupted research and publication processes. Consistent with previous literature [5–7, 13], the majority of studies in our cohort (90.4%) were published within the first three years after presentation, after which the likelihood of subsequent publication appeared to decline substantially.

Previous studies have suggested that the country in which a study is conducted may influence the likelihood of subsequent publication [1, 4, 13, 14]. Publication rates may reflect not only the scientific quality of the research but also the academic environment of the originating country [36]. Analyses from ECR and ESGAR meetings have consistently shown higher publication rates among Western European countries such as Switzerland, the Netherlands, France, and Germany, while several Asian countries, including South Korea and Japan, have demonstrated notable increases in publication output over time [1, 4, 6, 13]. In the present study, the highest publication rates were observed in the Netherlands, South Korea, and Germany. Previous research has indicated that English proficiency and national research funding levels are important determinants of publication output in high-impact medical journals [37]. The Netherlands serves as a prime example in this context, having ranked first for six consecutive years in the EF English Proficiency Index [38]. This international assessment of English skills among non-native speakers demonstrates the country’s consistently strong English proficiency. These factors may partially explain the higher publication rates observed in certain countries, although differences in research culture, institutional support, and topic selection are also likely to contribute.

Prospective studies have been consistently reported to achieve higher publication rates than retrospective ones across the radiology and medical literature [3, 22, 36, 39]. In the analysis of ECR 2010, although the overall publication percentages of prospective and retrospective abstracts were comparable, prospective studies tended to be published in higher-impact journals [1]. Interestingly, Secil et al., in their evaluation of ESGAR abstracts, reported the opposite trend, finding that retrospective studies were published more frequently than prospective ones, with a statistically significant difference [6]. In our study, no significant difference was observed between prospective and retrospective designs. It is difficult to determine the underlying causes of the inconsistent results observed across different meetings, as numerous factors may influence the overall process of transforming a presentation into a publication. In addition, studies categorized as “Other” showed markedly lower publication rates compared with both of these groups. This group mainly included experimental, methodological, or technical reports, as well as abstracts with unspecified designs. Such studies are generally less likely to be developed into full-length research articles.

In this study, both national and international collaborations demonstrated significantly higher publication rates compared with locally conducted studies. Articles arising from international collaborations received significantly higher citation counts. Although the mean journal impact factor was also higher in this group, the difference did not reach statistical significance. Similar trends have been reported in previous literature. Analyses from the ECR 2010 showed that authors engaged in international collaborations achieved higher publication rates and published in journals with greater impact factors [1]. Studies from other medical fields have likewise shown that multicenter or collaborative research tends to have higher publication success compared with single-center studies [40]. A large-scale analysis involving 60 institutions from multiple continents also demonstrated that international collaboration was associated with increased citation counts [41].

Collaborations across institutions and countries may enhance research quality by combining diverse expertise and optimizing available resources. Such partnerships can broaden the scientific perspective, strengthen study design, and improve the clarity of interpretation. The exchange of ideas during this process often functions as an informal internal review, allowing potential weaknesses to be identified and addressed before journal submission. These factors may increase the likelihood that collaborative studies result in well-developed manuscripts with greater visibility and academic impact.

In our study, publication rates differed significantly across subspecialties. Abstracts related to brain imaging showed the highest likelihood of subsequent publication, whereas those focused on spine and pediatric neuroradiology demonstrated lower rates. This may, in part, reflect the rapid evolution of advanced imaging techniques specific to brain research, such as functional magnetic resonance imaging (MRI), diffusion tensor imaging, and ultra–high-field MRI [42–45]. The relatively higher publication rate observed in brain-related studies may be associated with several field-specific factors, including the pace of technological innovation and the overall intensity of research activity in this area. Conversely, pediatric studies have also been reported to show lower publication rates in other congresses [13, 22]. Pediatric neuroimaging procedures present several challenges during image acquisition, including the need for sedation or anesthesia and the presence of motion artifacts [46]. In addition, patient populations are relatively smaller than in adults. These factors may partly contribute to the difficulties of conducting research in this subgroup. Beyond these technical aspects, economic factors may also influence publication outcomes. For instance, analyses of the National Institutes of Health (NIH) funding priorities have shown that within the neurosciences, the largest proportion of research grants is allocated to neuro-oncology, whereas spine research receives the lowest level of funding [47]. Overall, the variation in publication outcomes across subspecialties likely reflects a combination of methodological, technical, and field-specific factors. However, these interpretations should be considered exploratory and interpreted with caution. Further research is warranted to better elucidate the underlying causes of these differences.

This study has several limitations. First, the publication search was restricted to the MEDLINE database via PubMed. While this may have excluded some publications indexed elsewhere, PubMed provides a standardized reference point and has been consistently used in previous studies, thereby allowing reliable comparisons. Second, only oral abstracts were included, whereas poster presentations were excluded. This choice ensured comparability with most previous studies that primarily focused on oral presentations; however, it is possible that some poster abstracts also resulted in full-text publications. In addition, subspecialty classification was based on the congress labels provided in the official abstract booklets, which were largely consistent across years but may still represent a potential source of minor misclassification. Third, despite the use of a systematic search strategy, there remains a possibility that some publications were overlooked. In addition, citation counts were recorded as of October 15, 2025. Because citation accumulation is a dynamic process, this may introduce a time-related bias, with older publications having had more opportunity to accrue citations than more recent ones. To mitigate this effect, annualized citation rates were also calculated; however, this approach cannot fully account for variations in early versus long-term citation dynamics. Google Scholar was chosen as the source because of its broad coverage and free accessibility without the need for institutional subscriptions; however, it is not a selective database and may include inconsistencies and higher citation counts compared with curated databases, which should be considered as a limitation. Another limitation is that only full-text original research articles were considered, while case reports, reviews, technical notes, and letters were excluded, possibly underestimating overall publication rates. Finally, only abstracts from 2018 to 2022 were included, as previous studies have shown that the majority of publications occur within the first three years after presentation [6, 13, 14]. Nevertheless, some presentations, particularly those from 2021 to 2022, may still be published beyond the study’s follow-up period, which could result in an underestimation of publication rates for the later congresses.

Despite these limitations, this study provides important insights into the publication patterns of research presented at ESNR, representing the first investigation focusing on this congress. By employing a methodology consistent with previous investigations of other major radiology meetings, the study enables meaningful comparison across different subspecialty congresses. Moreover, it contributes to a better understanding of academic productivity and scientific impact associated with one of the leading international meetings in neuroradiology. Encouraging international collaboration may further enhance the visibility and impact of future studies presented at the meeting. In addition, future initiatives aimed at supporting research development, mentorship, and funding opportunities could be beneficial for fostering academic engagement within the ESNR framework. Strengthening such support mechanisms may ultimately contribute to sustained growth in research output and quality across the neuroradiology community.

## Conclusion

In this study, 47.3% of oral presentations at ESNR congresses were subsequently published in peer-reviewed journals. The findings reveal that multi-institutional collaboration plays a key role in publication success, with nationally and internationally collaborative studies achieving significantly higher rates than single-center research. Moving forward, ESNR may benefit from initiatives that actively promote collaborative networks, provide mentorship for early-career researchers, and offer structured support for manuscript development. Strengthening these efforts may help enhance the quality, visibility, and scientific impact of future research within the field of neuroradiology.

## Data Availability

No datasets were generated or analysed during the current study.
